# Supplementary figures and images for: eDNA captures depth partitioning in a kelp forest ecosystem
Source: PLoS One. 2021 Nov 4;16(11):e0253104. doi: 10.1371/journal.pone.0253104 (PMC8568143; doi:10.1371/journal.pone.0253104)

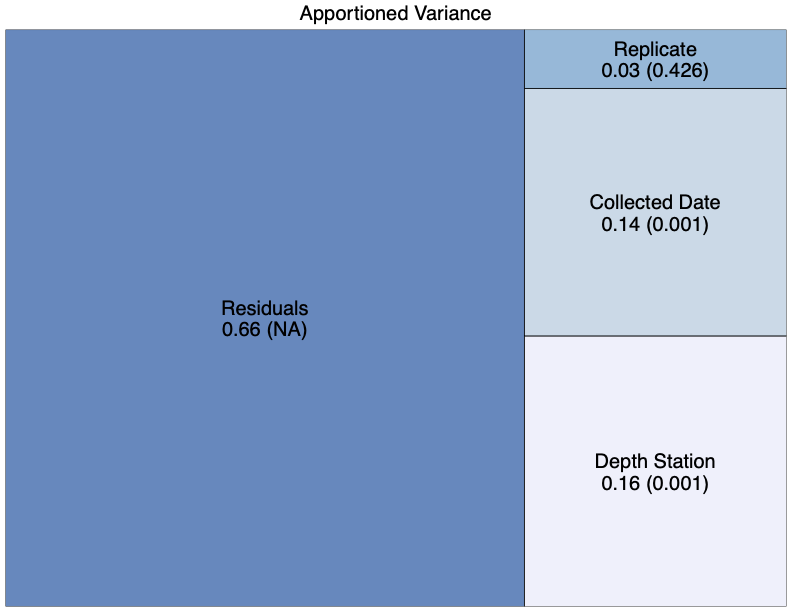

Supplement: S1 Fig — Depth accounted for 16% variance (p = 0.001), collected date accounted for 14% variance (p = 0.001) and replicate accounted for 3% variance (p = 0.426). (TIFF) [file pone.0253104.s001.tiff]

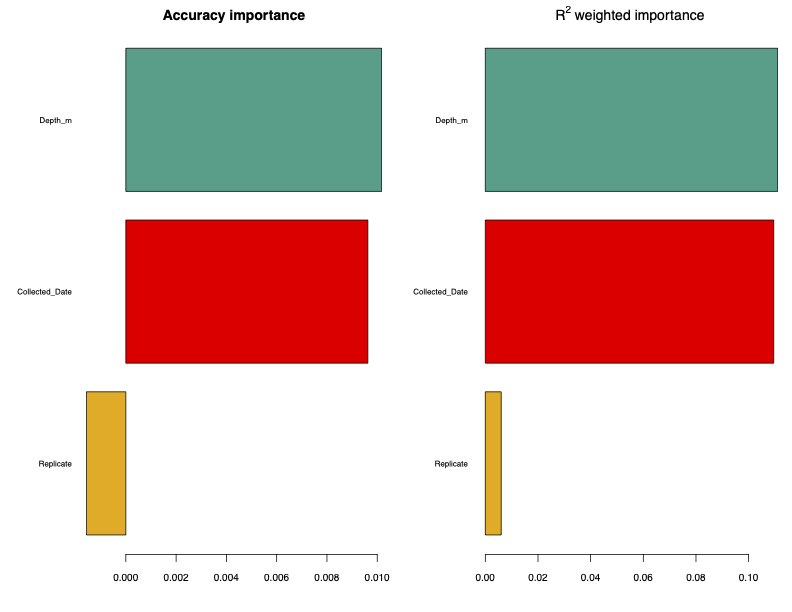

Supplement: S2 Fig — Sampling depth had the highest accuracy importance and R2 weighted importance in the depth gradient model. (TIFF) [file pone.0253104.s002.tiff]

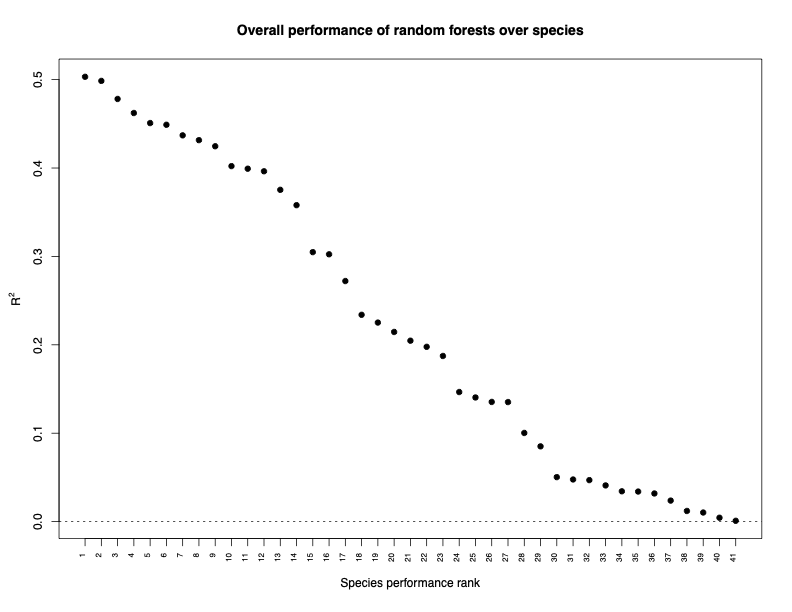

Supplement: S3 Fig — There were fourteen top predictor species with R2 values greater than 0.35 in the gradient forest model. (TIFF) [file pone.0253104.s003.tiff]

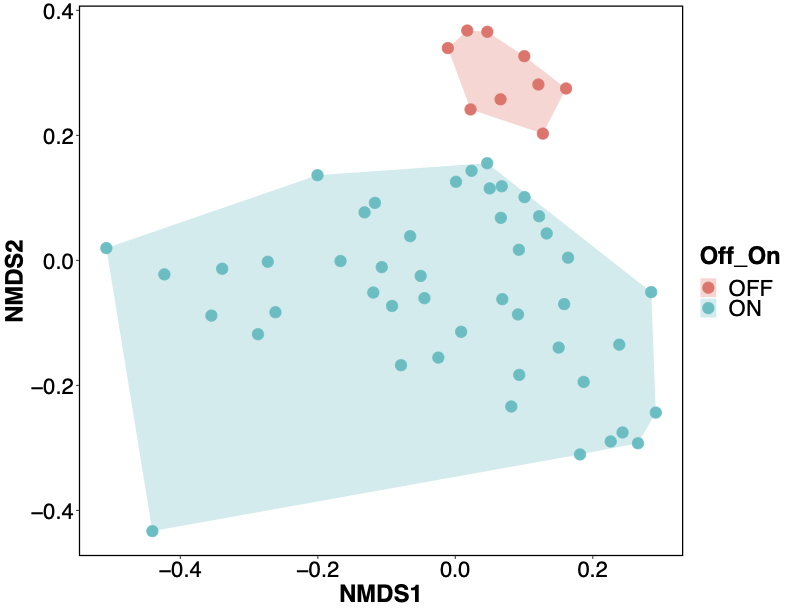

Supplement: S4 Fig — Surf zone community composition differs from the nearshore community composition. NMDS stress is 0.082. (TIFF) [file pone.0253104.s004.tiff]

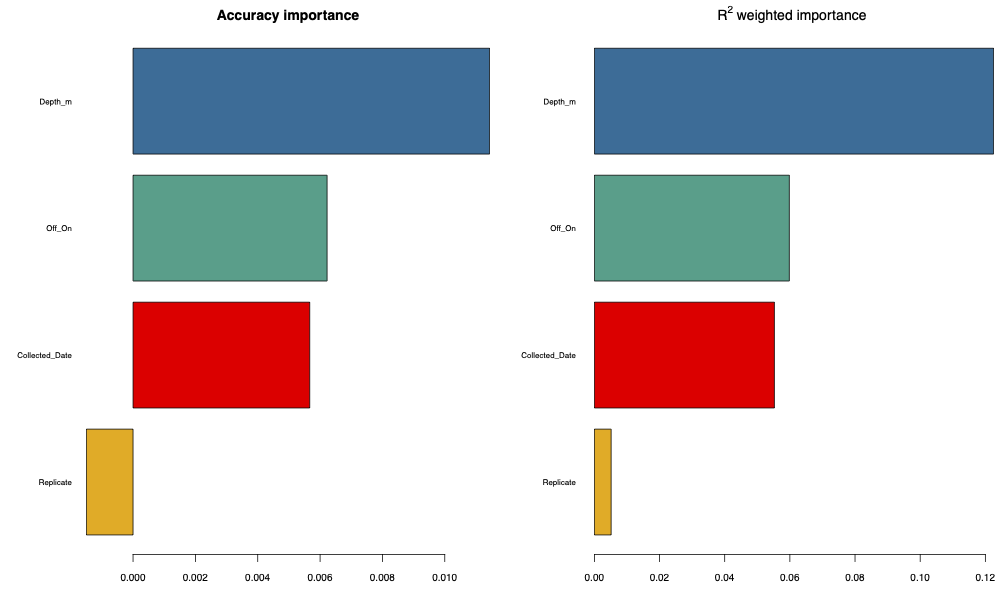

Supplement: S5 Fig — Sampling depth had the highest accuracy importance and R2 weighted importance in the nearshore vs. surf zone space gradient forest model. (TIFF) [file pone.0253104.s005.tiff]

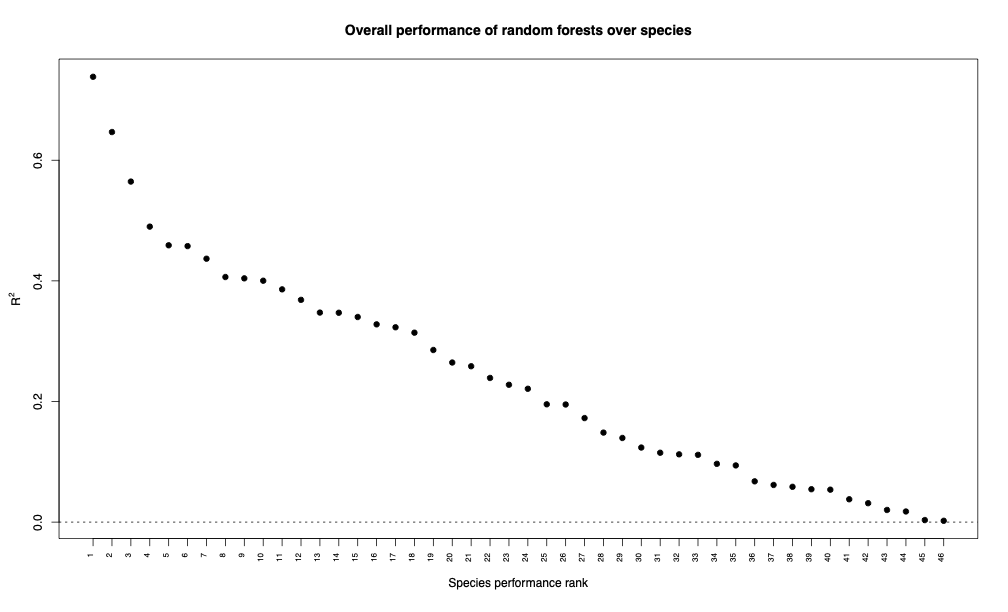

Supplement: S6 Fig — There were seven top predictor species with R2 values greater than 0.40 in the gradient forest model. (TIFF) [file pone.0253104.s006.tiff]
