# Supplementary material for: eDNA captures depth partitioning in a kelp forest ecosystem
Source: PLoS One. 2021 Nov 4;16(11):e0253104. doi: 10.1371/journal.pone.0253104 (PMC8568143; doi:10.1371/journal.pone.0253104)
Supplement: S1 Appendix — (PDF) [file pone.0253104.s014.pdf]

S1 Appendix. The extracted DNA volume for each sample was 100 µL. For PCR reactions with MiFish-U primers, we amplified eDNA in 25 µL reactions utilizing 12.5 µL Qiagen Multiplex Taq PCR 2x Master Mix, 6.5 µL dH20, 2.5µL of each primer (2 mM), and 1 µL template DNA. However, for all MiFish-E PCR reactions, we used 1.5x primer concentration to improve amplification, reducing water to maintain a 25uL reaction volume. Thermocycling employed a touchdown PCR profile consisting of: an initial denaturation at 95 ˚C for 15 minutes, 13 denaturation cycles at 94 ˚C for 30 seconds each, beginning annealing at 69.5 ˚C for 30 seconds with every subsequent cycle temperature decreasing by 1.5 ˚C until reaching 50 ˚C, extension at 72 ˚C for 1 minute with 35 additional cycles at annealing temperature 50 ˚C and final extension at 72 ˚C for 10 minutes. We ran each PCR in triplicate, and used both positive and negative PCR controls.

We determine the success of each PCR reaction through electrophoresis on a 2% agarose gel. We ran gels for 25 minutes on 150 volts, prior to viewing on a Dark Reader Transilluminator. We then pooled 5 µL from each sample triplicate PCRs for a total volume of 15 µL for each amplified eDNA sample. We then cleaned pooled samples with Serapure magnetic beads [1] and quantified DNA concentrations using the BR Assay Kit (Thermofisher Scientific, Waltham, MA, USA) on a Victor3 plate reader (Perkin Elmer, Waltham, MA, USA). Next, we attached barcodes to the samples using the IDT for Illumina Nextera Unique Dual Indexes (Illumina, San Diego, CA, USA) [2]. We indexed the MiFish-U and MiFish-E PCR products from the same sample with the same index, except for one sample with different indices for the MiFish-U and MiFish-E PCR products (sample Day2_10m_A). Indexing reactions consisted of 25 µL reaction mixture with 12.5 µL Kapa HiFi Hotstart Ready Mix (Kapa Biosystems, Wilmington, MA, USA), 1.25 µL Nextera UD Index, and 5 ng of DNA. The indexing PCR used the following PCR

thermocycler profile: initial denaturation at 95 ˚C for 5 minutes, 5 cycles of denaturation at 98 ˚C for 20 seconds, annealing at 56 ˚C for 30 seconds, extension at 72 ˚C for 3 minutes, and final extension at 72 ˚C for 5 minutes. We confirmed correct size of indexed PCR products through electrophoresis on a 2% agarose gel. We cleaned and quantified appropriately sized samples using the aforementioned bead cleaning protocol, and then pooled even copy numbers for each marker (MiFish-U and MiFish-E) across samples to ensure the DNA concentration was consistent across barcodes.

References

1. Faircloth BC, Glenn TC. Protocol: Preparation of an AMPure XP substitute (AKA Serapure). 2014. doi:10, J9MW2F26.

2. MacConaill LE, Burns RT, Nag A, Coleman HA, Slevin MK, Giorda K, et al. Unique, dual-indexed sequencing adapters with UMIs effectively eliminate index cross-talk and significantly improve sensitivity of massively parallel sequencing. BMC Genomics. 2018;19: 30. doi:10.1186/s12864-017-4428-5
