# Supplementary material for: eDNA captures depth partitioning in a kelp forest ecosystem
Source: PLoS One. 2021 Nov 4;16(11):e0253104. doi: 10.1371/journal.pone.0253104 (PMC8568143; doi:10.1371/journal.pone.0253104)
Supplement: S2 Appendix — (PDF) [file pone.0253104.s015.pdf]

S2 Appendix. We implemented a decontamination pipeline developed by Ryan Kelly and Ramón Gallego [1]. The first step estimates index hopping, or how often an index "jumps" across samples, which can lead to incorrect sample assignment [2]. To control for index hopping, we characterized the sequences in the positive controls and subtracted those sequences from all the environmental samples. The second step discards poorly sequenced samples, which can arise from poor library pooling. We discard all the sequences that fall below the 95% threshold of the normal distribution for total sequences. The third step removes contamination from positive and negative controls by removing the ASVs that occur most frequently in positive and negative controls. For each ASV, we calculate the maximum proportion, mean proportion, total number of reads, and prevalence of reads in all samples. If all the statistics are higher in either the positive or environmental samples, we label the ASV as a control or environmental sequence. If the statistics conflict, we only remove the ASVs that have maximum abundance in the positive controls. After the third step, we save the output for use in the alpha diversity comparisons. We use the output from this step, before the site occupancy modeling, to include as many possible rare taxa as possible for a more complete species richness analysis.

The last decontamination step is the site occupancy modeling, which characterizes if an ASV is true rare sequence or a likely contaminant. As with all site occupancy models, we assume occupancy state does not change between sampling efforts and that detections at a site are completely independent [3]. Whether a site is occupied (z=1) or not (z=0) can be evaluated using a Bernoulli trial with probability of occupancy (z ~ Bern(w)). The occupancy probability is constant within a site, which is incorporated in the model through a logit-linear model. The binomial parameter of conditional on site occupancy status was defined as P[i] <- z[i] x P11 + (1-z[i]) x P10 1) where the probability of species occurrences at a location denoted PSI 2) the

conditional probability of species occurrence within an eDNA sample from a site given that the species was truly at the site (true positive detection) denoted PS11 3) the conditional probability of a species occurrence within an eDNA sample from a site given that the species was falsely at the site (false positive detection) denoted PS10. The probability occurrence function used was the following:

Probability of Occurrence = (PSI x (P11^N) x (1-P11)^(K-N)) / ((PSI x (P11^N) x (1-P11)^(K- N))+(((1-PSI) x (P10^N)) x ((1-P10)^(K-N))))

Where K is the number of samples taken within a site and N is the number of ASV detections within a site.

Occupancy probability (PSI) and true positive probability (P11) were modeled with uninformative priors between 0 and 1. False positive probability (P10) was modeled with priors from a left-skewed beta distribution alpha = 1 and beta = 20. Each ASV has a presence-absence to feed the model. We removed all ASVs which had a site occupancy probability of less than 80%. We used this output for beta diversity statistics to measure community differences.

References

1. Kelly RP, Gallego R, Jacobs-Palmer E. The effect of tides on nearshore environmental DNA. PeerJ. 2018;6:e4521. doi:DOI 10.7717/peerj.4521

2. Costello M, Fleharty M, Abreu J, Farjoun Y, Ferriera S, Holmes L, et al. Characterization and remediation of sample index swaps by non-redundant dual indexing on massively parallel sequencing platforms. BMC Genomics. 2018;19: 332. doi:10.1186/s12864-018-4703-0

3. Lahoz-Monfort JJ, Guillera-Arroita G, Tingley R. Statistical approaches to account for false-positive errors in environmental DNA samples. Molecular Ecology Resources. 2016;16: 673–685. doi:10.1111/1755-0998.12486
